# Development of a new assessment scale for measuring interaction during staff-assisted transfer of residents in dementia special care units

**DOI:** 10.1186/s12877-015-0003-6

**Published:** 2015-02-10

**Authors:** Charlotta Thunborg, Petra von Heideken Wågert, Eva Götell, Ann-Britt Ivarsson, Anne Söderlund

**Affiliations:** 1 School of Health, Care and Social Welfare Mälardalen University, Västerås, Sweden; 2 School of Health and Medical Sciences, Örebro University, Örebro, Sweden

**Keywords:** Caregiver, Dementia, Dyadic interaction, Observation scale, Scale construction, Special care unit

## Abstract

**Background:**

Mobility problems and cognitive deficits related to transferring or moving persons suffering from dementia are associated with dependency. Physical assistance provided by staff is an important component of residents’ maintenance of mobility in dementia care facilities. Unfortunately, hands-on assistance during transfers is also a source of confusion in persons with dementia, as well as a source of strain in the caregiver. The bidirectional effect of actions in a dementia care dyad involved in transfer is complicated to evaluate. This study aimed to develop an assessment scale for measuring actions related to transferring persons with dementia by dementia care dyads.

**Methods:**

This study was performed in four phases and guided by the framework of the biopsychosocial model and the approach presented by Social Cognitive Theory. These frameworks provided a starting point for understanding reciprocal effects in dyadic interaction. The four phases were 1) a literature review identifying existing assessment scales; 2) analyses of video-recorded transfer of persons with dementia for further generation of items, 3) computing the item content validity index of the 93 proposed items by 15 experts; and 4) expert opinion on the response scale and feasibility testing of the new assessment scale by video observation of the transfer situations.

**Results:**

The development process resulted in a 17-item scale with a seven-point response scale. The scale consists of two sections. One section is related to transfer-related actions (e.g., capability of communication, motor skills performance, and cognitive functioning) of the person with dementia. The other section addresses the caregivers’ facilitative actions (e.g., preparedness of transfer aids, interactional skills, and means of communication and interaction). The literature review and video recordings provided ideas for the item pool. Expert opinion decreased the number of items by relevance ratings and qualitative feedback. No further development of items was performed after feasibility testing of the scale.

**Conclusions:**

To enable assessment of transfer-related actions in dementia care dyads, our new scale shows potential for bridging the gap in this area. Results from this study could provide health care professionals working in dementia care facilities with a useful tool for assessing transfer-related actions.

**Electronic supplementary material:**

The online version of this article (doi:10.1186/s12877-015-0003-6) contains supplementary material, which is available to authorized users.

## Background

Moving about in the environment is one of the most important activities of daily living. People with advanced dementia commonly experience considerable cognitive impairment and often have impaired motor skills [1]. This impairment interferes with their ability to independently change their position from lying, to sitting or standing [2]. This in turn affects their ability to start and complete their daily activities. Overall, 89% of residents with dementia living in long term care facilities are reported to have mobility limitations [1]. Mobility problems negatively affect the residents’ health and well-being because they are prone to falling, pressure ulcers, and pneumonia [3,4].

Caregivers are frequently required to assist people with advanced dementia execute physical transfers [5]. Providing hands-on assistance involves a complex interaction of personal factors (e.g., communicative effectiveness and self-efficacy) and physical abilities of the person with dementia and the caregiver, as well as environmental factors (e.g., furniture and transfer aids). Among these numerous factors, dyadic interaction related to physical transfers places a large burden on both individuals in the care dyad [6,7] .Therefore, care for mobility limitations needs to reflect residents’ characteristics, as well caregivers’ characteristics and environmental factors.

The mutual effect of transfer-related actions that are carried out by dementia care dyads can be described in terms of different “behavioral cues” presented by each individual. The term “behavior” is usually used to refer to behaviors of concern according to the Behavioral and Psychological Symptoms of Dementia consensus statement [8] when discussing people with dementia. Overall, an action is a part of behavior. To relate behavioral cues to a transfer situation, these actions can be described in terms of how the caregiver introduces a transfer to the person with dementia. In most cases, the actions involve helping the person with dementia to understand what to do by providing verbal and non-verbal cues and hands-on assistance [6]. If actions of the caregiver are ambiguous, the person with dementia might show signs of physical responses (e.g., grabbing, holding, or gripping the chair) [5]. This in turn creates pressure on the caregiver and mutual misunderstanding within the care dyad [7]. An assessment scale is required for describing and quantifying the effect of actions on performance of care dyads for transferring persons with dementia.

Numerous performance-based assessment scales have been developed because an important part of this transfer is the evaluation process of functional capabilities in dementia [9]. There are 17 common assessment scales for recording activities of daily living in individuals with dementia [10]. Additionally, the use of global rating scales [11-14] is relevant for evaluation of dementia. The Neuropsychiatric inventory [15] and the Cohen Mansfield Agitation index are frequently used in the clinic [16]. The aim of global rating scales is different from that of performance-based scales. Generally, global rating scales provide information regarding overall functioning of a problematic area. These scales do not take into account environmental factors, mobility-related actions, and the mutual influence within the care dyad [17]. Another important aspect is that a scale needs to address the caregivers’ characteristics (e.g., caregivers’ skills in providing person-centered care [18], assessing abusive behavior [19], or communication skills [20]. Nevertheless, specific assessment scales addressing hands-on assistance during transferring persons with dementia and interaction within a care dyad in dementia care facilities have not been developed.

To design a new scale that focuses on an individual’s transfer-related actions, the biopsychosocial model [21] along with the Social Cognitive Theory (SCT) [22] can provide such an approach. The SCT describes the mutual exchange between an individual’s actions (i.e., behavior of the care dyad), the contextual environment, and the individual’s personal characteristics (e.g., care dyad’s cognition, interactional skills, communication, and functional ability) [23].

A new scale is needed to examine care dyads’ actions related to problems of transfer in dementia care facilities. Therefore, this study aimed to develop an assessment scale for measuring actions during staff-assisted transfers involving residents and caregivers in dementia care facilities.

## Methods

Different steps need to be undertaken to develop an assessment scale [24]. In this study, four phases were performed, based on studies by Mahoney et al. [25] and Fayers and Machin [26]. First, for item generation, we examined the scientific literature regarding existing assessment scales. Second, video-recorded situations of transferring persons with dementia were collected and analyzed to provide ideas for the items. Third, the first author created an item pool and experts (registered physiotherapists) rated the relevance of proposed items in daily transfer of persons with dementia in special care units. Finally, we needed to choose a response scale of the final assessment scale. We drafted and tested the final scale for feasibility in transferring persons with dementia by observation with video.

### Ethical considerations

The Regional Ethical Review Board in Uppsala, Sweden (dnr 2012/146 and dnr 2009/359) approved the study. If a person with dementia demonstrated discomfort or aversion, the video recording was terminated. Proxy consent was gathered from the person’s next of kin by asking “Do you oppose that your next of kin participate?” The next of kin was also provided with written and oral information on the study. Caregivers participating in the study were also provided with oral and written information and they were asked to sign the written and informed consent to participate.

### Phase 1. Literature review for item generation

The purpose of the literature review was to identify existing assessment scales that are related to transfer-related actions of care dyads in terms of biopsychosocial factors of interest for item generation.

We conducted purposive sampling through examination of the current scientific literature, regulatory guidelines, and clinical practice guidelines. The first author performed a review of the literature during November 2012 to March 2013. The following databases were used: PubMed, Psych Info, CINAHL, and Google Scholar. Keywords included dementia care, assessment scale, and daily activity. Moreover, for the caregivers’ field, the combined keywords were measurement, dementia caregiving, and interaction. Only assessment scales based on empirical studies, written in English, and published from January 1980 to 2013, were included.

### Phase 2. Video-recorded situations of transferring persons with dementia for item generation

The purpose of video observation was to capture the actions of dementia care dyads as they moved persons with dementia. Video also enabled interpretation of actions to create items.

Video recordings of transferring persons with dementia (n = 7) were gathered by the first author. Non-participant observations were used as the principle method. Two cameras allowed each transfer situation to be viewed from two different angles. Brief logbook notes complemented video-recorded data that were not captured by the films (e.g., if another caregiver or resident was entering the room). Inclusion of transfer situations was consecutively performed. For the video recordings, residents (n = 3) whom staff identified as having mobility problems when they needed to be transferred were chosen according to Lawton et al. [1] (i.e., being on one’s feet, changing position, and changing location) (see Table 1 for sample characteristics).

**Table 1:** **Sample characteristics of video observations**

Characteristics	Caregivers (n = 10 )	Persons with dementia (n = 3)
Age, years, mean (range)	43 (30–58)	83 (78–90)
MMSE, mean (range) *(0–30)		4 (0–12)
**Sex**		
Male	1	2
Female	9	1
**Profession**		
Nurse’s assistant	8	
Nurse aides	2	

MMSE: Mini-mental state examination. *Less than or equal to 23 points indicates cognitive impairment.

Three persons with dementia and 10 professional caregivers gave their informed consent to participate in the video observations. Additionally, we gathered proxy consent from the person with dementia’s next of kin. The first author performed the Mini Mental State Examination [27]. Video-recorded sessions were as follows: 1) awaken, get out of bed, and walk to the day room (n = 1); 2) awaken, get out of bed, and walk to the toilet (n = 3); 3) get out of a chair, walk, and get into an armchair (n = 1); 4) get out of bed, walk, and get into a wheelchair (n = 1); and 5) get out of a chair in the day room, walk back to one’s own apartment, and lie down on the bed (n = 1). The first author performed the analysis according to biopsychosocial aspects of transferring persons.

### Phase 3. Creating the item pool and rating the relevance of proposed items

The purpose of phase three was to create the item pool, rate the relevance of each item, and choose the items for the final assessment scale.

The first author generated the item pool through the results from phases one and two. Analysis performed by the research team resulted in an item pool consisting of 93 items. Of the 93 items, 51 items were related to actions of the person with dementia and 42 were related to the caregivers’ actions in situations of assistance during transferring persons with dementia. In the next step, the item pool was sent by e-mail to 54 experts (registered physiotherapists). The experts were asked to rate the relevance of each item on a 4-point Likert scale (1 = not relevant, 2 = somewhat relevant, 3 = quite relevant, and 4 = highly relevant). They were also asked to provide detailed qualitative comments regarding the item pool. Fifteen experts chose to participate. A modified Delphi process [28,29] containing one round was used. The first author reviewed and analyzed all submitted ratings and comments from the experts. Analyses of the scores from the relevance ratings were performed by computing the Item Content Validity Index (Item-CVI) [30] with IBM statistical software SPSS Statistics for Windows, version 19.0 (Armonk, NY: IBM Corp). The relevance rating score in relation to the number of experts when there are more than five experts must be at least 0.83. We chose the lowest relevance score of 0.93 for inclusion of items in the assessment scale, which means that 14/15 experts scored the item as relevant or highly relevant. Abstraction of qualitative feedback from the experts was performed by analyzing and carefully deliberating all comments for suggestions of construction of items, an additional file shows this in more detail (see Additional file 1).

### Phase 4. Expert opinions on the response scale and feasibility testing of the assessment scale

The purpose of the last phase was to identify the appropriate response scale for the assessment scale, and to test the new scale for feasibility in situations of helping transfer a person with dementia.

The response scale and the number of points in the assessment scale were discussed in one meeting with six research colleagues. Four PhD-level students and two professors who were within the area of health, care and welfare, took part in the discussion. They discussed the pros and cons of two different response scales (i.e., the 0–10 Numeric Rating Scale [31] and the 7-point Likert scale [32]), as well as interpretation of the assessment scale.

In the feasibility test, different situations of helping to move persons with dementia were included. Transfer situations were randomized from an earlier data collection (video observations). The first author performed the test by observing and scoring the video-recorded transfer situations by using the new scale. In total, eight care dyads consisting of eight persons with dementia and nine caregivers were included. Inclusion criteria of care dyads were based on observation of three characteristics of mobility problems in persons with dementia. These characteristics were being on one’s feet, changing position, and changing location [1]. The feasibility test should provide information to the research team regarding the usability of the assessment scale and uncover any misfits in item construction or scaling responses.

## Results

The results are arranged in the order in which the development process was performed. In order to provide the results of each phase with a context, some explanations of the results are provided in the text.

### Phase 1

We did not find any papers that addressed assessment scales in the specific topic of interest (i.e., transfer-related actions of dementia care dyads). Nevertheless, a total of 42 papers were examined on the relevance for covering biopsychosocial aspects for item generation addressing dyadic interaction in transfer of persons with dementia. In total, 15 papers were included. The papers addressed the following: scales of activities of daily living (e.g., characteristics associated with limitations in mobility) [33-37], cognitive disability (e.g., characteristics associated with the understanding of the particular situation of transfer) [38-40], and communication difficulties (e.g., verbal and non-verbal cues) in individuals with dementia disease [41-44]. Assessment scales that addressed characteristics of caregivers’ actions in daily caregiving situations were also included for item generation [20,45]. These included verbal and non-verbal caregiver strategies to facilitate communication and understanding in persons with dementia [46].

### Phase 2

The video recordings captured verbal and non-verbal actions and cues. These represented items addressing communication strategies of the caregiver, as well as actions representing transfer-related interactions in the care dyad. An example of one of these items was that actions addressing discomfort were expressed as verbal complaints and non-verbal cues (e.g., clenched teeth and rocking) of the person with dementia. This in turn gave input to items related to that type of action in the person with dementia. Information of who participated in the transfer (one or two caregivers) and which of the caregivers directed the interaction were recognized as important factors. Watching movement cycles beginning with a clear observable or audible caregiver action or prompt directed towards the person with dementia to engage in the transfer situation gave ideas for items addressing caregivers’ different actions to trigger motor activity. Furthermore, ideas that covered actions emphasizing the extent to which the transfer situations were prepared by the caregiver were generated (e.g., if a walker or a wheelchair were ready to be used).

**Figure 1:**
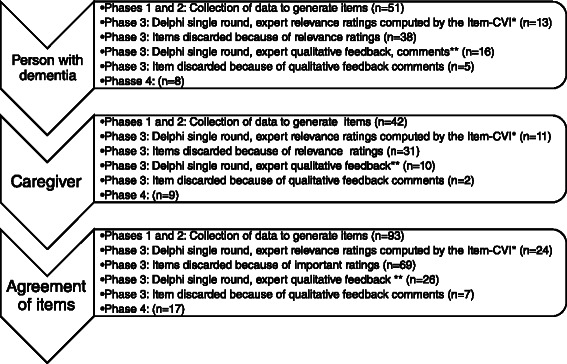
**Flowchart for the generation process (phases 1–4) of items (n).** Phases 1–4 describing the inclusion and exclusion process of items of persons with dementia, the caregivers, and agreement of the items. Exact item generation process see Additional file 1. *Experts participating (n = 15); **experts providing comments (n = 7).

Analysis of relevance of items as computed with the Item-CVI resulted in inclusion of 24 items. For further refinement, double negation in some of the items was removed by reformulation. The qualitative feedback and opinions on wording used to describe the constructs in the items are described in the Additional file 1. According to the comments from the experts, there was a need for generalization and specification of some items, as problems of expression were identified (see Additional file 1). To gain clarity, this process resulted in a 17-item assessment scale. The first eight items cover the actions of persons with dementia and the next nine items cover the caregivers’ actions in situations of helping transfer a person with dementia, see Figure 1 for further description of the generation of items.

### Phase 3

Expert panel discussions resulted in a seven-point response scale for the new scale, with anchor points of one (optimal) and seven (non-optimal). The expert discussions covering the interpretation of the new assessment scale resulted in a multi-item scale with a total score that is separate for persons with dementia and caregivers, as well as individual item scores. The summary score for each area indicates the possible presence of problematic interactional transfer-related actions of the care dyad from a biopsychosocial perspective. The range in score of the person with dementia area can be 8–56 and that of the caregiver can be 9–63 points. Lower scores indicate a higher ability to perform interactional transfer-related actions in both areas (see Table 2 for further description of items).

**Table 2:** **Description of items**

DIDTAS – Items for persons with dementia (PWDs)	
1.	PWD is able to remain attentive in the transfer situation
2.	PWD is able to actively participate in the transfer situation
3.	PWD has goal-orientated movement patterns in the transfer situation
4.	PWD moves at a goal-orientated tempo
5.	PWD has bodily control in relation to his/her surroundings
6.	PWD does not express discomfort through body language in the transfer situation
7.	PWD does not express discomfort through words/sounds in the transfer situation
8.	PWD is independent in the transfer situation
**DIDTAS – Items for caregivers**	
9	Caregiver provides instructions for transfer just before beginning transfer
10.	Caregiver provides a clear verbal command about transfer
11.	Request for transfer is followed by the caregiver waiting for a PWD to respond
12.	If two caregivers are present, one of them assists with cooperation of the PWD □ Not applicable
13.	Transfer situations are performed in a safe manner for the PWD
14.	Caregiver adapts their actions to facilitate the transfer situation of the PWD
15.	Caregiver maintains contact with the PWD during the transfer situation
16.	Transfer aids are available before the start of the transfer situation
17.	Interaction with the PWD is optimal for the transfer situation

DIDTAS: Dyadic interaction in dementia transfer assessment scale.

### Phase 4

Identification and scoring of all items were found to be satisfying. However, we observed that the two discomfort items could be part of the pain-related actions of individuals with dementia. Rating of all 17 items for every video took between 1 and 3 minutes, with a total of approximately 20 minutes for all videos. Interaction is bidirectional by nature. Therefore, sometimes actions of the care dyad were intertwined, which made them difficult to identify and rate. To orient the items, the person with dementias’ and the caregivers’ area was managed at the same page. Transfer aids were present in all transfer situations. Therefore, actions related to the item of *transfer aids available before the start of transfer* were easy to observe and rate. Generally, the feasibility test showed that transfer-related actions presented by dementia care dyads could be identified and scored, with no obvious redundancy of items.

## Discussion

This study resulted in a new 17-item assessment scale that measures dementia care dyads’ transfer-related actions during staff-assisted transfer situations at special care units. To the best of our knowledge, no other scale can assess the bidirectional influence of transfer-related actions created by care dyads. An earlier review of Alzheimer’s disease scales stated that assessment criteria of cognition, communication, behavior and activity of daily living were the most clinical relevant areas to be included in a new scale [10]. Additional endpoints considered as clinical relevant as well were patient’s quality of life and caregiver burden. Except for the latter two, the newly developed assessment scale concern these areas, which can be considered as a strength.

For physiotherapists and occupational therapists working in dementia care facilities, our new scale provides proxy reporting by observation on transfer of persons. When developing a new scale, describing the raters’ special educational requirements, as well as the features of the population under consideration, is important [26]. In this study, the intended users’ initial training or special educational requirements were used to assess motor skills and transfer-related actions of dementia care dyads.

According to Fayers and Machin [26], development of a new assessment scale should follow specific sequences, and each of the nine suggested phases should be documented thoroughly. In the current study, the four phases included a total of seven steps out of the nine recommended steps [26], which can be considered as a strength.

The first author of this study performed a literature review of previously published scales, as recommended by several authors [24,26,47]. If two independent reviewers had assessed the scales for suitability and quality, this would have increased the quality of the present study. However, the literature review was guided by the biopsychosocial framework and assessment scales in related areas that contributed to ideas of item generation. Examining related areas concerning similar problems is recommended [24], and this could be considered as a strength of our study. In our study, the fields of neuropsychological disability [48,49] autism disorders [50], and interaction-communication with infants [51] provided a useful contribution to addressing dyadic interaction.

In the current study, the theoretical framework of the biopsychosocial model and the features of SCT provided a basis for development of a new scale that addresses transfer-related actions in dementia care dyads. The utility of a theoretical framework and its application in development of an assessment scale has been debated. DeVellis [47] asserted that a theory plays a major role in conceptualization of the measurement problem. This in turn, means that the researcher needs detailed knowledge on the phenomena and the abstract relationship that exists to identify items consistent with a theory. However, Worthington and Whittaker [52] stated that the theoretical and the rational and logical approaches are no longer popular methods in development of scales. Instead, the empirical approach, which also uses statistical analysis (e.g., confirmatory factor analysis of item responses), is the preferred model to form homogenous item groupings [26]. In this study, confirmatory factor analysis was not applicable because of the low amount of data from video observations. This is a limitation to the study. However, the development process guided by a theoretical approach and confirmatory factor analysis approach are not mutually exclusive. In the future, a study needs to be designed for factor analysis as a recommended part of domain structure and construct validity.

Video observation in our study offered the potential for a detailed analysis and information regarding actions related to “here and now” (e.g., non-verbal actions related to caregivers’ hands-on assistance). Determining the exact expression of each action can be difficult. However, with video recording, manifestation of actions could be viewed on numerous occasions, which increased the chance to identify actions that could ease construction of items. The first author performed the analysis, aiming to identify aspects of transfer-related actions that could be interpreted within the biopsychosocial framework. However, no predefined categories were identified, which is a limitation to this study.

There are two main issues when conducting video observations in dementia care facilities. One issue is the phenomena of “subject reactivity” [53]. The other issue is the highly important ethical consideration about issues that may arise when seeking informed consent from persons with dementia to be video-recorded. With regard to subject reactivity, there were too few video observations for each dementia care dyad to investigate the unawareness of the subjects who were participating. This is a limitation and might have caused bias to the item generation process. However, the unpredictability of each care dyad’s transfer actions is often too complex to have time to direct one’s behavior in another direction. Therefore, in this study, we did not know if subject reactivity was a problem. With regard to ethical considerations, we cannot always be certain that a person with dementia really understands what he or she is agreeing to. However, it is possible to determine when they no longer want to participate. As proposed by Heggestad et al. [54], moral sensitivity guided the whole process in the present study when video recording individuals with dementia.

The expert review of proposed items was to build-up criterion validity of the scale [26] and is important for the scale development process [55]. Unfortunately, only one round of the modified Delphi technique [47] was used in this study. However, the high level of expertise of the 15 experts providing qualitative feedback contributed to reducing the ambiguity of items. Furthermore, the Item-CVI, which computed the relevance of each item [30], resulted in a cut-off point (0.93) for inclusion of items (i.e., 14 of 15 experts rated the item as relevant or very relevant), which is a strength of this study.

Construct and face validity could have been evaluated if the recommended step of patient interviews [26] had been performed. Unfortunately, this step was not applicable in this study because of the cognitive deficits of individuals with dementia living in special care units. However, interviews of caregivers might have helped take the caregivers’ concerns into consideration. Therefore, not performing caregiver interviews could be a limitation of this study.

The choice of a seven-point response scale with anchor points of one and seven offers the rater sufficient points of discrimination to be sensitive to changes, without having to maintain too many response options in mind (i.e., compared with the 0–10 response scale) [24]. This is a strength of our study, which can increase the usability of the assessment scale.

The feasibility test provided evidence that all items could be identified and rated, and no further revision was needed. Feasibility testing is one of the most important steps in the development process of an assessment scale [26,24]. However, discussing the first author’s involvement in the feasibility test is important for bias of the results. We cannot prove that the items of our assessment scale are a valid operationalization of the construct or that the constructs are a valid representation of actions [26].

Future methodological steps should address the reliability and validity of our scale. Investigation of inter-and intra-rater reliability need to be undertaken before the scale is used in research and/or in clinical practice. Additionally, future studies should aim to investigate the sensitivity and whether constructs of items behave in line with expectations.

## Conclusion

This study reported the development of a new assessment scale that measures transfer-related actions of dementia care dyads. The development process, including four phases, was iterative and comprehensive, using the biopsychosocial model and the approach presented by the SCT as guidance. A literature review and video recordings were used for ideas for the item pool. Item-CVI and expert qualitative feedback reduced the number of items. The feasibility test showed that all items could be identified and rated. Therefore, our assessment scale could be useful for physiotherapists and occupational therapists in their clinical work of assessing situations of helping transfer persons with dementia in special care units. Our study makes a significant contribution to bridging the gap in the area of assessment of transfer-related actions of care dyads.

## Additional file

Additional file 1:
**This table shows a presentation of suggestions for items for constructing items in the final assessment scale.**

## References

[1] Williams SW, Williams CS, Zimmerman S, Sloane PD, Preisser JS, Boustani M (2005). Characteristics associated with mobility limitation in long-term care residents with dementia. Gerontologist.

[2] Oddy R (2003). Promoting Mobility for People with Dementia: A Problem-Solving Approach.

[3] Creditor MC (1993). Hazards of hospitalization of the elderly. Ann Intern Med.

[4] Harper CM, Lyles YM (1988). Physiology and complications of bed rest. J Am Geriatr Soc.

[5] Varnam W (2011). How to mobilise patients with dementia to a standing position. Nurs Older People.

[6] Thunborg C, von Heideken Wågert P, Söderlund A, Götell E (2012). Reciprocal struggling in person transfer tasks – caregivers’ experiences in dementia care. Adv Phys.

[7] Wångblad C, Ekblad M, Wijk H, Ivanoff SD (2009). Experiences of physical strain during person transfer situations in dementia care units. Scand J Caring Sci.

[8] Finkel SI, Costa E, Silva J, Cohen G, Miller S, Sartorius N (1996). Behavioral and psychological signs and symptoms of dementia: a consensus statement on current knowledge and implications for research and treatment. Int Psychogeriatr.

[9] Gitlin LN, Roth DL, Burgio LD, Loewenstein DA, Winter L, Nichols L (2005). Caregiver appraisals of functional dependence in individuals with dementia and associated caregiver upset: psychometric properties of a new scale and response patterns by caregiver and care recipient characteristics. J Aging Health.

[10] Robert P, Ferris S, Gauthier S, Ihl R, Winblad B, Tennigkeit F (2010). Review of Alzheimer’s disease scales: is there a need for a new multi-domain scale for therapy evaluation in medical practice?. Alzheimers Res Ther.

[11] Reisberg B, Ferris SH, de Leon MJ, Crook T (1988). Global Deterioration Scale (GDS). Psychopharmacol Bull.

[12] Krzyminska E, Rossa G, Krzyminski S (1993). The Global Deterioration Scale (GDS) and Functional Assessment Staging (FAST) in the diagnosis of Alzheimer type dementia. Psychiatr Pol.

[13] Chang YL, Bondi MW, McEvoy LK, Fennema-Notestine C, Salmon DP, Galasko D (2011). Global clinical dementia rating of 0.5 in MCI masks variability related to level of function. Neurology.

[14] Baumgarten M, Becker R, Gauthier S (1990). Validity and reliability of the dementia behavior disturbance scale. J Am Geriatr Soc.

[15] Cummings JL, Mega M, Gray K, Rosenberg-Thompson S, Carusi DA, Gornbein J (1994). The Neuropsychiatric Inventory: comprehensive assessment of psychopathology in dementia. Neurology.

[16] Cohen-Mansfield J, Billig N (1986). Agitated behaviors in the elderly. I A Conceptual Rev J Am Geriatr Soc.

[17] Bliwise DL, Lee KA (1993). Development of an Agitated Behavior Rating Scale for discrete temporal observations. J Nurs Meas.

[18] Gaugler JE, Hobday JV, Savik K (2013). The CARES((R)) observational tool: a valid and reliable instrument to assess person-centered dementia care. Geriatr Nurs.

[19] Cooper C, Dow B, Hay S, Livingston D, Livingston G (2013). Care workers’ abusive behavior to residents in care homes: a qualitative study of types of abuse, barriers, and facilitators to good care and development of an instrument for reporting of abuse anonymously. Int Psychogeriatr.

[20] Williams CL, Parker C (2012). Development of an observer rating scale for caregiver communication in persons with Alzheimer’s disease. Issues Ment Health Nurs.

[21] Engel GL (1981). The clinical application of the biopsychosocial model. J Med Philos.

[22] Bandura A (1977). Social Learning Theory. Prentice-Hall Series in Social Learning Theory.

[23] Bandura A (1989). Human agency in social cognitive theory. Am Psychol.

[24] Streiner DL, Norman GR (2008). Health Measurement Scales: A Practical Guide to their Development and Use.

[25] Mahoney EK, Hurley AC, Volicer L, Bell M, Gianotis P, Hartshorn M (1999). Development and testing of the Resistiveness to Care Scale. Res Nurs Health.

[26] Fayers PM, Machin D (2007). Quality of Life: The Assessment, Analysis, and Interpretation of Patient-Reported Outcomes. 2. rev. ed.

[27] Cockrell JR, Folstein MF (1988). Mini-Mental State Examination (MMSE). Psychopharmacol Bull.

[28] de Villiers MR, de Villiers PJ, Kent AP (2005). The Delphi technique in health sciences education research. Med Teach.

[29] Morgan PJ, Lam-McCulloch J, Herold-McIlroy J, Tarshis J (2007). Simulation performance checklist generation using the Delphi technique. Can J Anaesth.

[30] Polit DF, Beck CT, Owen SV (2007). Is the CVI an acceptable indicator of content validity? Appraisal and recommendations. Res Nurs Health.

[31] Jensen MP, Karoly P, Braver S (1986). The measurement of clinical pain intensity: a comparison of six methods. Pain.

[32] Thurstone LL (1954). The measurement of values. Psychol Rev.

[33] Lichtenstein MJ, Burger MC, Shields SL, Shiavi RG (1990). Comparison of biomechanics platform measures of balance and videotaped measures of gait with a clinical mobility scale in elderly women. J Gerontol.

[34] Aberg AC, Lindmark B, Lithell H (2003). Development and reliability of the General Motor Function Assessment Scale (GMF)–a performance-based measure of function-related dependence, pain and insecurity. Disabil Rehabil.

[35] Pan AW, Fisher AG (1994). The Assessment of Motor and Process Skills of persons with psychiatric disorders. Am J Occup Ther.

[36] Reisberg B (1988). Functional assessment staging (FAST). Psychopharmacol Bull.

[37] Mahurin RK, DeBettignies BH, Pirozzolo FJ (1991). Structured assessment of independent living skills: preliminary report of a performance measure of functional abilities in dementia. J Gerontol.

[38] Barberger-Gateau P, Commenges D, Gagnon M, Letenneur L, Sauvel C, Dartigues JF (1992). Instrumental activities of daily living as a screening tool for cognitive impairment and dementia in elderly community dwellers. J Am Geriatr Soc.

[39] Basic D, Rowland JT, Conforti DA, Vrantsidis F, Hill K, LoGiudice D (2009). The validity of the Rowland Universal Dementia Assessment Scale (RUDAS) in a multicultural cohort of community-dwelling older persons with early dementia. Alzheimer Dis Assoc Disord.

[40] Bobholz JH, Brandt J (1993). Assessment of cognitive impairment: relationship of the Dementia Rating Scale to the Mini-Mental State Examination. J Geriatr Psychiatry Neurol.

[41] Alexopoulos G, Abrams R, Young R, Shamoian C (1988). Cornell scale for depression in dementia. Biol Psychiatry.

[42] Hughes CP, Berg L, Danziger WL, Coben LA, Martin RL (1982). A new clinical scale for the staging of dementia. Br J Psychiatry.

[43] Wallin A, Edman A, Blennow K, Gottfries CG, Karlsson I, Regland B (1996). Stepwise comparative status analysis (STEP): a tool for identification of regional brain syndromes in dementia. J Geriatr Psychiatry Neurol.

[44] Hurley AC, Volicer BJ, Hanrahan PA, Houde S, Volicer L (1992). Assessment of discomfort in advanced Alzheimer patients. Res Nurs Health.

[45] Grosch K, Medvene L, Wolcott H (2008). Person-centered caregiving instruction for geriatric nursing assistant students: development and evaluation. J Gerontol Nurs.

[46] Campbell JL, Rowe MA, Marsiske M (2011). Behavioral symptoms of dementia: a dyadic effect of caregivers’ stress process?. Res Gerontol Nurs.

[47] DeVellis RF (2012). Scale Development: Theory and Applications. 3rd ed. Applied Social Research Methods Series.

[48] Friedman-Weieneth JL, Doctoroff GL, Harvey EA, Goldstein LH (2009). The Disruptive Behavior Rating Scale—Parent Version (DBRS-PV): factor analytic structure and validity among young preschool children. J Atten Disord.

[49] Gioia GA, Isquith PK, Guy SC, Kenworthy L (2000). Behavior rating inventory of executive function. Child Neuropsychol.

[50] Lord C, Risi S, Lambrecht L, Cook EH, Leventhal BL, DiLavore PC (2000). The autism diagnostic observation schedule-generic: a standard measure of social and communication deficits associated with the spectrum of autism. J Autism Dev Disord.

[51] Messinger DS, Mahoor MH, Chow S-M, Haltigan JD, Cadavid S, Cohn JF, Gratch J, Marsella S (2014). Early emotional communication: Novel approaches to interaction. Social Emotions in Nature and Artifact.

[52] Worthington RL, Whittaker TA (2006). Scale development research a content analysis and recommendations for best practices. Couns Psychol.

[53] Elder JH (1999). Videotaped behavioral observations: enhancing validity and reliability. Appl Nurs Res.

[54] Heggestad AK, Nortvedt P, Slettebo A (2013). The importance of moral sensitivity when including persons with dementia in qualitative research. Nurs Ethics.

[55] Cabrera-Nguyen P (2010). Author guidelines for reporting scale development and validation results. Soc Soc Work Res.

