# Supplementary material for: Development of a new assessment scale for measuring interaction during staff-assisted transfer of residents in dementia special care units
Source: BMC Geriatr. 2015 Feb 10;15:6. doi: 10.1186/s12877-015-0003-6 (PMC4333260; doi:10.1186/s12877-015-0003-6)
Supplement: Additional file 1: — This table shows a presentation of suggestions for items for constructing items in the final assessment scale. [file 12877_2015_3_MOESM1_ESM.pdf]

Additional file 1: This table shows a presentation of suggestions for items for constructing items in the final assessment scale					
**No.***	**Item**		**Expert comments**	**Explanation as to why items have been rephrased**	**Construction of items in the final assessment scale**
1	6	The person with dementia is able to independently move from their wheelchair to their bed	Exp7: If the person with dementia is sitting in a wheelchair, how likely is it that he/she is able to transfer into and out of it safely?<br>Exp8: I have some difficulty with the definition of the concept "independent" in all of the items. A clear definition is needed for it to be of relevance, otherwise it is too open to interpretation. Do we mean physical or cognitive independence, exclusive or inclusive initiative capacity etc.?	Construction of items related to independence (items 6, 10, 15, 16, 17, and 21) of the person with dementia were generalized because of expert (experts 2, 6, 7, and 8) comments on the fact that these items were open to interpretation. In the final assessment scale, construction of items related to independence of the person with dementia was generalized to *attention (cognitive independence and initiative capacity), how actively involved is the person with dementia?,* and to *what degree is the person with dementia independent (physical capacity) in the transfer situation*? The observer was asked to describe the transfer situation as a whole according to environmental factors and transfer aids used before assessing the transfer situation (see also comment from exp8 on item 61, on construction of what type of aids are appropriate in the transfer situation).	Item 8: The person with dementia is independent in the transfer situation.<br><br>Item 1: The person with dementia is able to remain attentive in the transfer situation.
2	10	The person with dementia is able to	Exp8: Does this mean from lying to sitting? This function is found in		

		independently sit on the edge of their bed	other items. Perhaps it is unnecessary to include it here.<br>Exp3: Lying to sitting, or which function...?		
3	15	The person with dementia can independently get up from a sitting position	Exp7: Is there a bed support aid mounted on the bed? Does this include from a raised bed/chair?		
4	16	The person with dementia can stand independently	Exp7: Perhaps a good idea to clarify that the person with dementia is standing still.		
5	17	The person with dementia can walk independently	Exp8: Needs to be mentioned whether the person is able to turn, even with support.		
6	21	The person with dementia performs the entire transfer independently	Exp2: Which transfer?<br>Exp6: Depends on the surroundings.		
7	32	The person with dementia actively participates in the transfer	Exp8: The term “actively participates” is too vague. This needs clarification.	Item 32 was clarified in accordance with expert comment no. 8 regarding vague terminology. The construction of “actively participates” was found to better describe the interactional behavior of the person with dementia. The items related to holding onto supports and resistance to the direction of the transfer (so-called gegenhalten) were generalized to	Item 2: The person with dementia is able to actively participate in the transfer situation.

				items of persons with dementia related to movement patterns, body control, and movement speed in the transfer situation.	
8	37	The person with dementia appears to expresses discomfort in the transfer	Exp7: Some people with dementia have lost their speech ability but are still independent during transfer.<br>Exp8: This is an important item because people with dementia often express fear in the transfer situation.<br>Exp8: The concept “appears to express” is vague. You should use the concept “shows signs of” instead.	Construction of the term of “discomfort” was further specified because of expert (7, 8) comments on the importance of discomfort, the loss of verbal language in the person with dementia, and the vague meaning of the item.	Item 6: The person with dementia expresses no discomfort through body language in the transfer situation.<br><br>Item 7: The person with dementia expresses no discomfort by using words/sounds in the transfer situation.
9	39	The person with dementia resists the movement of the transfer by gripping various supports	Exp7: Why does the person with dementia do this? Are they uncertain?<br>Exp8: Is it a question of the person gripping something specific or just the support?<br>When I read “support”, I have images of bed support aids or trapezes in my mind. However, when someone grips		Item 3: The person with dementia has a goal-orientated movement pattern in the transfer situation.

			something, it can be anything in the vicinity – which one do you mean here?		
10	40	The person with dementia leans in the opposite direction to the transfer situation			Item 4: The person with dementia performs the transfer at a goal-orientated tempo.
11	42	The person with dementia cannot evaluate the distance to place where they will sit	Exp3: Items 42 and 43 are similar and I prefer a more general expression.		Item 5: The person with dementia has bodily control in relation to their surroundings.
12	43	The person with dementia sits down before having reached the place where they will sit	Exp3: See previous comment.		
13	47	The person with dementia needs verbal guidance during the entire transfer	Exp2: The combination of body language and oral exhortation is important.		
14	53	Request for transfer is repeated if necessary	Exp5: Often it is not only a question of verbal guidance with dementia.	Expert comments on these three items 53, 54, and 55, were generalized to the construction of what type of behavior the caregiver adopts to increase understanding of the transfer situation of the person with dementia.	Item 9: Caregiver provides instructions for transfer just before beginning transfer.<br><br>Item 10: Caregiver provides a verbal command about the transfer.

15	54	Request for transfer is simplified if necessary	Exp5: See previous question.		
16	55	Request for transfer is expressed in clear words	Exp3: Using just words is not sufficient.		
17	56	Request for transfer is reinforced through body language	Exp7: Reinforced or clarified means the same thing to me.		Item 12: If two caregivers are present, one of them assists with cooperation of the person with dementia.
18	57	Request for transfer is followed by the caregiver waiting for the person with dementia	There were no comments for this item	The construction of this item was clarified by adding the statement *to react* because it involves a direct observation of the behavior to initiate or start the transfer activity in the person with dementia.	Item 15: The caregiver maintains contact with the person with dementia during the transfer situation.<br><br>Item 11: Request for transfer is followed by the caregiver waiting for the person with dementia.
19	58	Request for transfer is clarified through body language	Exp7: Same as item 56.	Construction of the items related to reinforcement and simplification (items 56 and 58) of the transfer situation were generalized to item 15 in the final scale: caregiver maintains contact with the person with dementia.	
20	61	Transfer aids available before the start of transfer	Exp8: Only one item is required for transfer aids as in the assessment design. You can add a box where the observer can check which transfer aid(s)	Construction of the item was generalized to regarding the preparatory work that was performed by the caregiver before the transfer situation occurred.	Item 16: Transfer aids are available before the start of the transfer situation.

			was/were used in the specific situation.		
21	62	The room is adapted for transfer	Exp3: This item could be difficult to word.<br>Exp8: Needs clarification as to what is meant by an “adapted room”. Does it mean that the room is specially equipped to facilitate transfer or does it mean that the room is simply practical?	The construction of this item was generalized to the overall description of the transfer situation regarding the context and what type of transfer aids were used in the situation.	
22	68	The transfer situation is performed in a safe manner	Exp8: Safe in what way(s)?	Construction of this item was clarified in terms of the person with dementia.	Item 13: The transfer situation is performed in a safe manner for the person with dementia.
23	69	The caregiver works calmly during the transfer	Exp7: This question would be more relevant if it were worded as “the caregiver can adapt to the needs of the person with dementia”<br>Exp8: What does this mean? To work calmly can also mean to take care of routine tasks without adapting to the needs of the person with dementia.	Construction of this item was clarified in terms of the person with dementia.	Item 14: The caregiver adapts their actions to facilitate the transfer situation for the person with dementia.

24	82	The caregiver can adapt the transfer to the individual needs of the person with dementia	There were no comments for this item.		Item 17: Interaction with the person with dementia is optimal for the transfer situation.

*Number of items included in phase three (expert relevance rating). Exp: expert.
